# Aberrant expression of JNK-associated leucine-zipper protein, JLP, promotes accelerated growth of ovarian cancer

**DOI:** 10.18632/oncotarget.12069

**Published:** 2016-09-16

**Authors:** Ji Hee Ha, Mingda Yan, Rohini Gomathinayagam, Muralidharan Jayaraman, Sanam Husain, Jinsong Liu, Priyabrata Mukherjee, E. Premkumar Reddy, Yong Sang Song, Danny N. Dhanasekaran

**Affiliations:** ^1^ Stephenson Cancer Center, The University of Oklahoma Health Sciences Center, Oklahoma City, OK 73104, USA; ^2^ Department of Cell Biology, The University of Oklahoma Health Sciences Center, Oklahoma City, OK 73104, USA; ^3^ Department of Pathology, The University of Oklahoma Health Sciences Center, Oklahoma City, OK 73104, USA; ^4^ The University of Texas MD Anderson Cancer Center, Houston, TX 77030, USA; ^5^ Icahn School of Medicine at Mount Sinai, New York, NY 10029, USA; ^6^ Cancer Research Institute, Seoul National University, College of Medicine, Seoul 151-921, Korea

**Keywords:** JLP, JNK, scaffold, ovarian cancer, SPAG9

## Abstract

Ovarian cancer is the most fatal gynecologic cancer with poor prognosis. Etiological factors underlying ovarian cancer genesis and progression are poorly understood. Previously, we have shown that JNK-associated Leucine zipper Protein (JLP), promotes oncogenic signaling. Investigating the role of JLP in ovarian cancer, our present study indicates that JLP is overexpressed in ovarian cancer tissue and ovarian cancer cells. Transient overexpression of JLP promotes proliferation and invasive migration of ovarian cancer cells. In addition, ectopic expression of JLP confers long-term survival and clonogenic potential to normal fallopian tube-derived epithelial cells. Coimmunoprecipitation and colocalization analyses demonstrate the *in vivo* interaction of JLP and JNK, which is stimulated by lysophosphatidic acid (LPA), an oncogenic lipid growth factor in ovarian cancer. We also show that LPA stimulates the translocation of JLP-JNK complex to the perinuclear region of SKOV3-ip cells. JLP-knockdown using shRNA abrogates LPA-stimulated activation of JNK as well as LPA-stimulated proliferation and invasive migration of SKOV3-ip cells. Studies using ovarian cancer xenograft mouse model indicate that the mice bearing JLP-silenced xenografts exhibits reduced tumor volume. Analysis of the xenograft tumor tissues indicate a reduction in the levels of JLP, JNK, phosphorylated-JNK, c-Jun and phosphorylated-c-Jun in JLP-silenced xenografts, thereby correlating the attenuated JLP-JNK signaling node with suppressed tumor growth. Thus, our results identify a critical role for JLP-signaling axis in ovarian cancer and provide evidence that targeting this signaling node could provide a new avenue for therapy.

## INTRODUCTION

MAP-kinase modules play a crucial role in regulating several critical aspects of cell growth and differentiation [1]. Several lines of evidence indicate that the accelerated-signaling and signaling-fidelity of the MAP-kinase modules are facilitated by specific scaffold proteins [1–4]. Scaffold proteins physically interact with the component kinases to provide an insulated physical platform for the transmission signals from one kinase to the other. Emerging evidence indicates that the aberrant expression of scaffold proteins could potentially enhance oncogenic signaling presumably through the hyper-activation of the associated kinase modules [5]. Our previous findings along with others have indicated the potential role of JNK-associated Leucine zipper Protein (JLP) in regulating cell growth [6–9]. However, its role in cancer genesis and/or progression remains largely unknown. JLP is encoded by *SPAG9* gene, which generates three splice variants namely, JLP (3,921 bp; 1307 amino acids), JIP4 (3426 bp; 1142 amino acids), and SPAG9 (2,268 bp; 766 amino acids) [10]. Of these splice variants, JLP is ubiquitously expressed and provide a scaffold function for both JNK and p38MAPK [6]. Several studies have reported the overexpression of *SPAG9* gene product in many cancers [11–15]. However, the use of antibodies that cross-react with all of the splice variants has raised a major concern regarding the true identity of oncogenic splice variant of *SPAG9*. Nevertheless, the finding that *SPAG9*-*JAK2* fusion gene that contains exon-26 of JLP predicts poor outcome in pediatric acute lymphoblastic leukemia patients establishes a prognostic role for JLP [16]. Potential tumor promoting role for JLP is further substantiated by the cBioPortal analysis of TCGA dataset of ovarian cancer tissue, which indicates that the increased expression of *SPAG9/JLP* correlates with a reduction in the disease free survival of ovarian cancer patients [17–19]. In addition, the observation that the activation of JNK-signaling predicts poor survival of ovarian cancer patients indirectly points to the potential role of JNK-interacting JLP in disease prognosis [20, 21]. In ovarian cancer, lysophosphatidic acid (LPA) has been characterized as a potent lipid growth factor that elicits both mitogenic and motogenic response and thus promotes ovarian cancer progression and intraperitoneal spread of the disease [22–24]. Based on our previous findings that JLP is involved in LPA-stimulated activation of JNK [7, 8], we hypothesized that the aberrant expression of JLP could promote tumorigenesis or tumor progression in ovarian cancer. This was tested in the present study using ovarian cancer cell lines including those representing high-grade serous ovarian carcinoma (HGSOC) and ovarian cancer xenografts.

Our results indicate that JLP is overexpressed in ovarian cancer tissue compared to adjacent normal ovarian tissue. Increased expression of JLP is also observed in a panel of ovarian cancer cells representing high-grade serous ovarian carcinoma. Ectopic overexpression of JLP stimulates the proliferation as well as the invasive migration of ovarian cancer cells. More interestingly, ectopic expression of JLP promotes long-term survival and clonogenicity in normal fallopian tube-derived epithelial cells. We also demonstrate that JLP physically interacts with JNK *in vivo* and this interaction is stimulated by LPA. Our results also indicate that JLP is critically required for LPA-stimulated activation of JNK as well as LPA-stimulated proliferation and invasive migration of ovarian cancer cells. Using the mouse xenograft ovarian cancer model, we establish that the silencing of JLP attenuates the activation of JNK signaling module in the tumor tissue along with a resultant reduction in tumor growth and intraperitoneal spread of the disease. Thus, our data presented here identifies, for the first time, a tumor-promoting role for JLP in ovarian cancer growth and progression.

## RESULTS

### Overexpression of JLP in ovarian cancer

**Figure 1:**
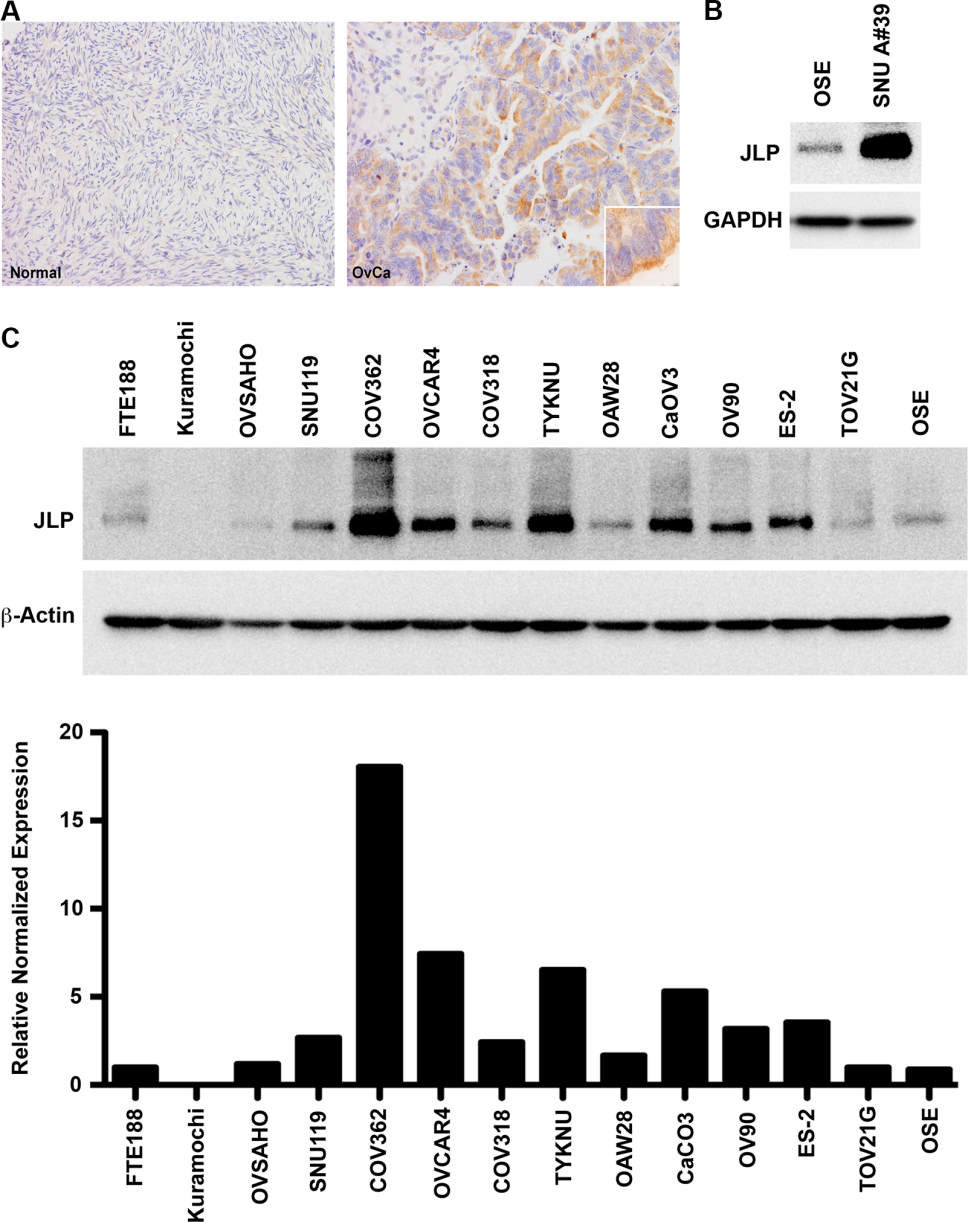
Expression of JLP in ovarian cancer (**A**) Expression of JLP in human ovarian cancer tissue compared to normal ovarian epithelial tissue. FFPE sections of normal ovarian tissue (*Left Panel, Normal*) and human ovarian cancer tissue (*Right Panel, OvCa*) was immunohistochemically stained against JLP. Magnification is 20X and the inset magnification is 60×. Typical image from five different patients is presented (*n* = 5). (**B**) Increased expression of JLP in patient-derived ovarian cancer cells. Immunoblot analysis was carried out using lysates from HGSOC patient derived ovarian cancer cells, SNU-A39, SNU-A5, and SNU-A8. Representative data from SNU-A39 is presented. OSE cell lysate was used as control. Stripped blot was probed with GAPDH to monitor equal protein loading. (**C**) Increased expression of JLP in HGSOC cells. Lysates from cell lines representing HGSOC were subjected to immunoblot analysis using JLP-antibodies. FTE188 and OSE cells were used as controls. The blot was stripped and probed for GAPDH to monitor equal protein-loading (Upper Panel) JLP-bands were quantified and normalized to GAPDH-expression levels (Lower Panel). Representative data from a typical experiment is presented (*n* = 3).

Our previous studies have indicated that JLP is required for JNK-mediated oncogenic signaling by the *gep*-family of oncogenes [7, 8]. Considering the determinant role of *gep* oncogenes and JNK-signaling in ovarian cancer progression, we investigated whether JLP shows increased expression in ovarian cancer tissues. Using antibodies that do not cross-react with JIP4 or SPAG9, we carried out an immunohistochemical analysis to monitor the expression of JLP in ovarian cancer tissue. As shown in Figure 1A, ovarian cancer tissue showed an increased expression of JLP compared to normal tissue. Increased expression of JLP could also be observed in ovarian cancer cells isolated from the ascites of patients (Figure 1B). Next we analyzed the expression of JLP in a panel of ovarian cancer cells representing HGSOC [25, 26]. Immortalized normal OSE and FTE188 cells were used as normal control cells. Results from immunoblot analyses indicated the overexpression of JLP in majority of the tested cell lines (10 out of the 12 tested cell lines) compared to the FTE or OSE cell lines (Figure 1C).

### Overexpression of JLP promotes oncogenic response

**Figure 2:**
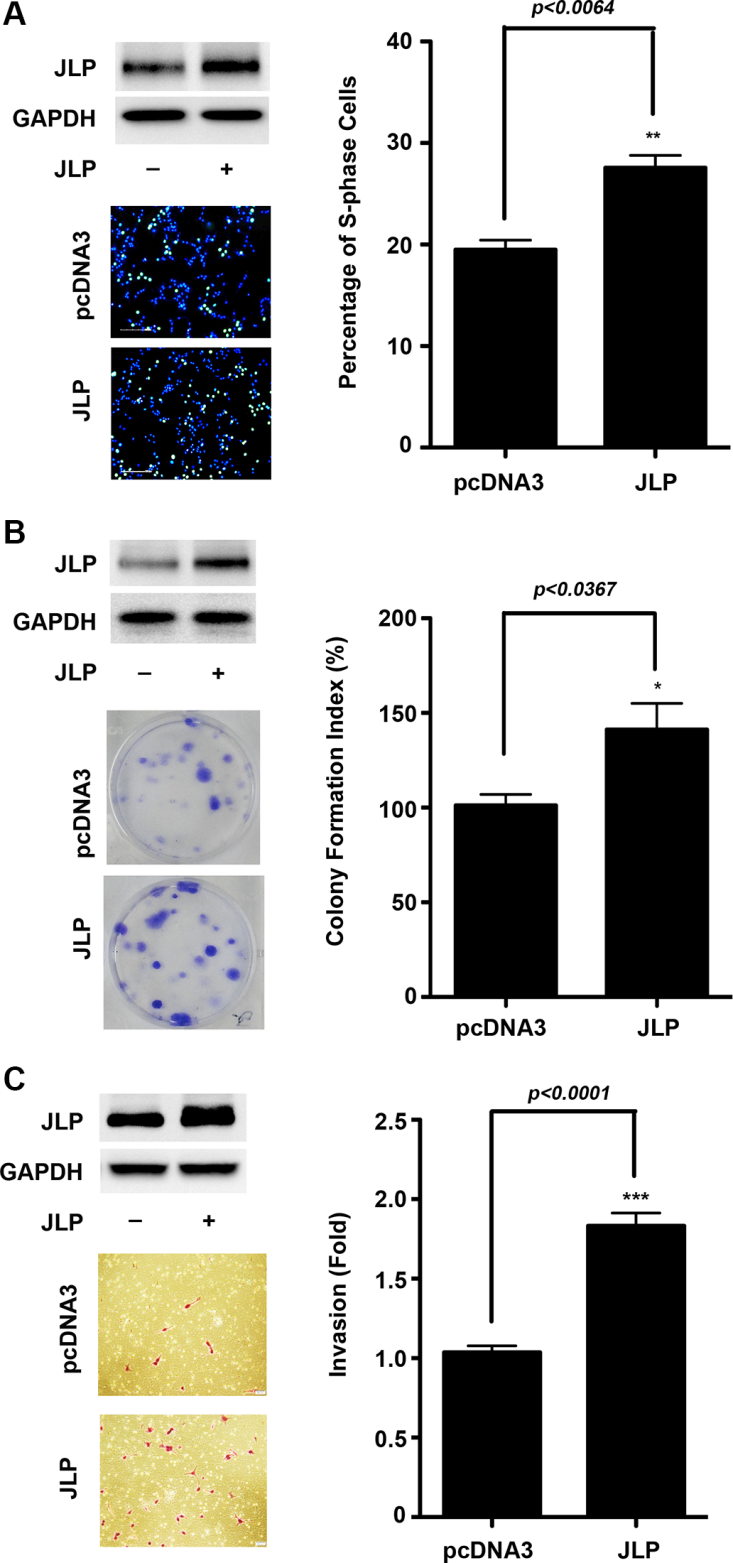
JLP stimulates proliferation and invasive migration (**A**) JLP stimulates cell proliferation. SKOV3-ip cells were transfected with vector encoding JLP or vector control. Proliferating S-phase cells were imaged using Click-iT Plus EdU assay as described in Supplementary Material 1. Proliferating cells (green) versus the total number of nuclear-stained cells (blue) were imaged (Left Panel) and quantified (Right Panel). Results are presented as percent of S-phase cells (Mean ± SEM; *n* = 3). (**B**) JLP stimulates the colony formation potential in normal fallopian tube derived epithelial cells. FTE188 cells were transfected with vector encoding JLP or empty vector. The transfectants were grown for 12 days and the colonies were visualized by staining with 0.2% crystal violet (Left Panel) and quantified using GelCount Colony Counter (Right Panel). Results represent Mean ± SEM (*n* = 3). (**C**) JLP enhances the invasive migration of SKOV3-ip cells. SKOV3-ip cells were transfected with vector encoding JLP or empty pcdNA3-vector. At 48 hrs, a transwell invasive migration was carried out as described previously [47]. Representative micrograph images of Hemacolor-stained invaded cells at 100× for each of the experimental groups are presented (Left Panel). Migrated cells were quantified and presented as fold change over untreated control values (Right Panel). Quantification is derived from three independent experiments (*n* = 3; Mean ± SEM).

Next, we investigated whether the increased expression of JLP contributes to mitogenic or motogenic responses of ovarian cancer cells. Mitogenic response was monitored using a cell proliferation assay that monitors the incorporation 5-ethynyl-2′-deoxyuridine (EdU), a fluorescent analog of thymidine, into DNA. Vector control SKOV3-ip cells along with cells in which JLP was overexpressed were stimulated with LPA and the S-phase cells that have incorporated EdU were imaged and quantified. As shown in Figure 2A, the overexpression of JLP stimulated a significant increase in the proliferation of ovarian cancer cells as indicated by 50% increase in EdU-incorporated cells. To further assess the potential of JLP on cell proliferation, we investigated whether the overexpression of JLP could contribute to the neoplastic transformation of the normal fallopian tube-derived epithelial cells. This was carried out using a modified clonogenic assay that assesses the long-term growth-promoting role of candidate genes [27]. As shown in Figure 2B, our results indicated that the overexpression of JLP promoted the increase in the number of SKOV3-ip colonies by 40% (101 ± 12 versus 142 ± 27 colonies per plate for the control and JLP respectively), thus validating the growth-promoting role of JLP. Next, we examined whether the overexpression of JLP confers invasive migratory phenotype to SKOV3-ip ovarian cancer cells. Invasive migrations of these cells were monitored using collagen-coated transwell assay. As shown in Figure 2C, results from this assay clearly indicated that the overexpression of JLP enhanced the invasive migration of SKOV3-ip cells by 80 % (12 ± 3 versus 23 ± 5 invaded cells per field for the vector control and JLP groups respectively).

### JLP-JNK interactions in ovarian cancer cells

**Figure 3:**
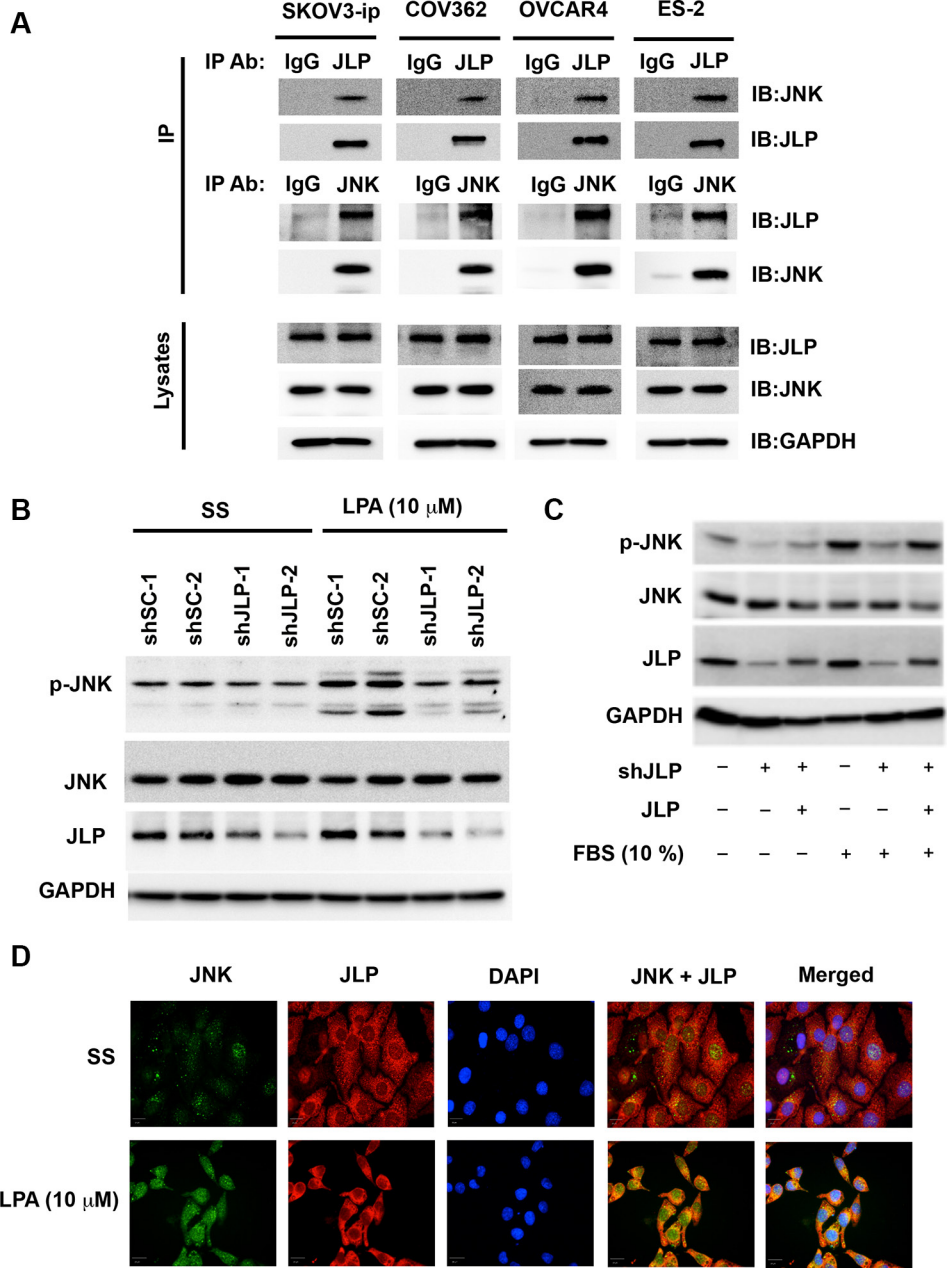
JLP-JNK interaction in ovarian cancer cell lines (**A**) JLP physically associates with JNK *in vivo*. Co-immunoprecipitation analyses were carried with the lysates derived from SKOV3-ip, OVCAR4, COV362 or ES-2 using antibodies to JLP (IP Panel: Upper Row) or JNK (IP Panel: Lower Row) along with IgG control. Immunoprecipitates, resolved by SDS-PAGE, were subjected to immunoblot analyses as indicated. Expression levels of JLP and JNK were monitored by immunoblot analyses using the respective antibodies (Lysates Panel). Equal protein-loading was monitored by reprobing the blots with GAPDH-antibodies. (**B**) JLP knockdown attenuates LPA-stimulated activation of JNK. Two independent clones of SKOV3-ip cells in which the expression of JLP was silenced by JLP-specific shRNAs (shJLP-1 and shJLP-2) and cells expressing control scrambled shRNAs (shSC-1 and shSC-2) were stimulated with LPA for 20 minutes. Lysates from these cells were analyzed for the activation of JNK in an immunoblot assay using antibodies that recognize phosphorylated Thr183/Thr185 of JNK. The blot was probed for the expression levels of JLP, JNK, and GAPDH using the respective antibodies. GAPDH-levels were used to monitor equal protein-loading. Results from a typical experiment are presented (*n* = 3). (**C**) Effect of knockdown of endogenous JLP and rescue by the transient expression of murine JLP on LPA-mediated JNK-activation. SKOV3-ip cells in which the expression of JLP was silenced using specific shRNA was transfected with shJLP-resistant murine JLP or vector control for 48 hrs. The transfectants were serum-starved for 6 hrs and stimulated with LPA for 20 minutes. Lysates from these cells were analyzed for the activation of JNK by immunoblot analysis for phospho-JNK. The blot was stripped and re-probed for JLP, JNK, and GAPDH. Results from a representative experiment are presented (*n* = 3). (**D**) LPA stimulates the co-localization of JNK and JLP in ovarian cancer cells. SKOV3-ip cells were serum-starved and stimulated with 10 μM LPA for 24 hours. Cells were fixed, immune-stained with murine monoclonal JNK-antibodies as well as rabbit-JLP-antibodies, labeled with anti-mouse Alexa 488- (green) and anti-rabbit Alexa 568- (red) fluorophore-conjugated secondary antibodies and counterstained with DAPI. Colocalization was assessed using Operetta high-content imaging system.

Previous studies have shown that JLP provides a molecular scaffold for the JNK signaling module and this can be monitored by the interaction between JLP and JNK [6–8]. The observation that the ovarian cancer cells show an increased expression of JLP prompted us to investigate whether JLP interacts with JNK in ovarian cancer cells. Co-immunoprecipitation analyses were carried out using lysates derived from SKOV3-ip cells in which JLP was transiently expressed for 48 hrs. As shown in Figure 3A, JNK can be observed along with the immunoprecipitated JLP in these cells. A reverse experiment in which JNK was immunoprecipitated showed the presence of JLP in JNK-Immunoprecipitates. Next, we investigated this further using COV362, OVCAR4, and ES2 cells that represent HGSOC [25]. As shown in Figure 3A, the results were similar to those obtained with SKOV3-ip cells. Together, these results demonstrated the endogenous interaction between JLP and JNK, further validating the scaffolding role of JLP *in vivo*. Functional analysis of JLP has shown that the interaction between JLP and JNK-module leads to the activation of JNK and subsequent translocation of activated JNK to nuclear periphery from where JNK can enter into the nucleus activating specific transcription factors including c-Jun [6]. Our previous studies have shown that LPA stimulates the interaction between JLP and JNK and this interaction leads to the activation of JNK [7, 8]. Therefore, in the present study, we sought to investigate whether the physical interaction seen with JLP and JNK is translated into the activation of JNK. This was analyzed by testing whether the knockdown of JLP could abrogate LPA-stimulated activation of JNK in SKOV3-ip ovarian cancer cells. SKOV3-ip cells in which the expression of JLP was knocked down using specific shRNA and control cells expressing scrambled shRNA were stimulated with 10 μM LPA. As shown in Figure 3B, two independent clones in which the expression of JLP was silenced showed a reduction in the phosphorylated JNK levels. Quantification of the data indicated that the knockdown of JLP attenuated LPA-stimulated activation of JNK (68 ± 11%; Mean ± SD). To further confirm that the reduction in JNK-activity is due to the silencing of JLP, we tested the overexpression of murine JLP, which is resistant to the shRNA used in this study, could rescue the shJLP-mediated attenuation of JNK-activity. Our results indicated that shJLP reduced the activation of JNK by 81% whereas the murine JLP could in fact rescue the attenuating effect of shJLP (80% recovery), thus further establishing the role of JLP in JNK-activation (Figure 3C). Our previous studies have shown that the interaction of JLP with JNK leads to the translocation of the JLP-JNK complex to nuclear periphery from where the phosphorylated JNK could enter into nucleus. This was examined by JLP-JNK colocalization analysis using fluorescence microscopy. Serum starved SKOV3-ip cells were stimulated with 10 μM LPA for 24 hrs. Cells were fixed and immunostained for JNK and JLP using Alexa Fluor 488 (green)- and Alexa Fluor 568 (red)-labeled antibodies respectively. Our results indicated that JLP and JNK showed a diffused localization throughout the cytosol with minimal colocalization in the absence of LPA (Figure 3D, Upper Panel). However, following stimulation with 10 μM LPA, an increased colocalization of JLP and JNK in the nuclear periphery could be observed (Figure 3D, Lower Panel). Together, these results point to the critical role of JLP in LPA-mediated activation of JNK in ovarian cancer cells.

### Role of JLP in LPA induced proliferation and invasive migration of ovarian cancer cells

**Figure 4:**
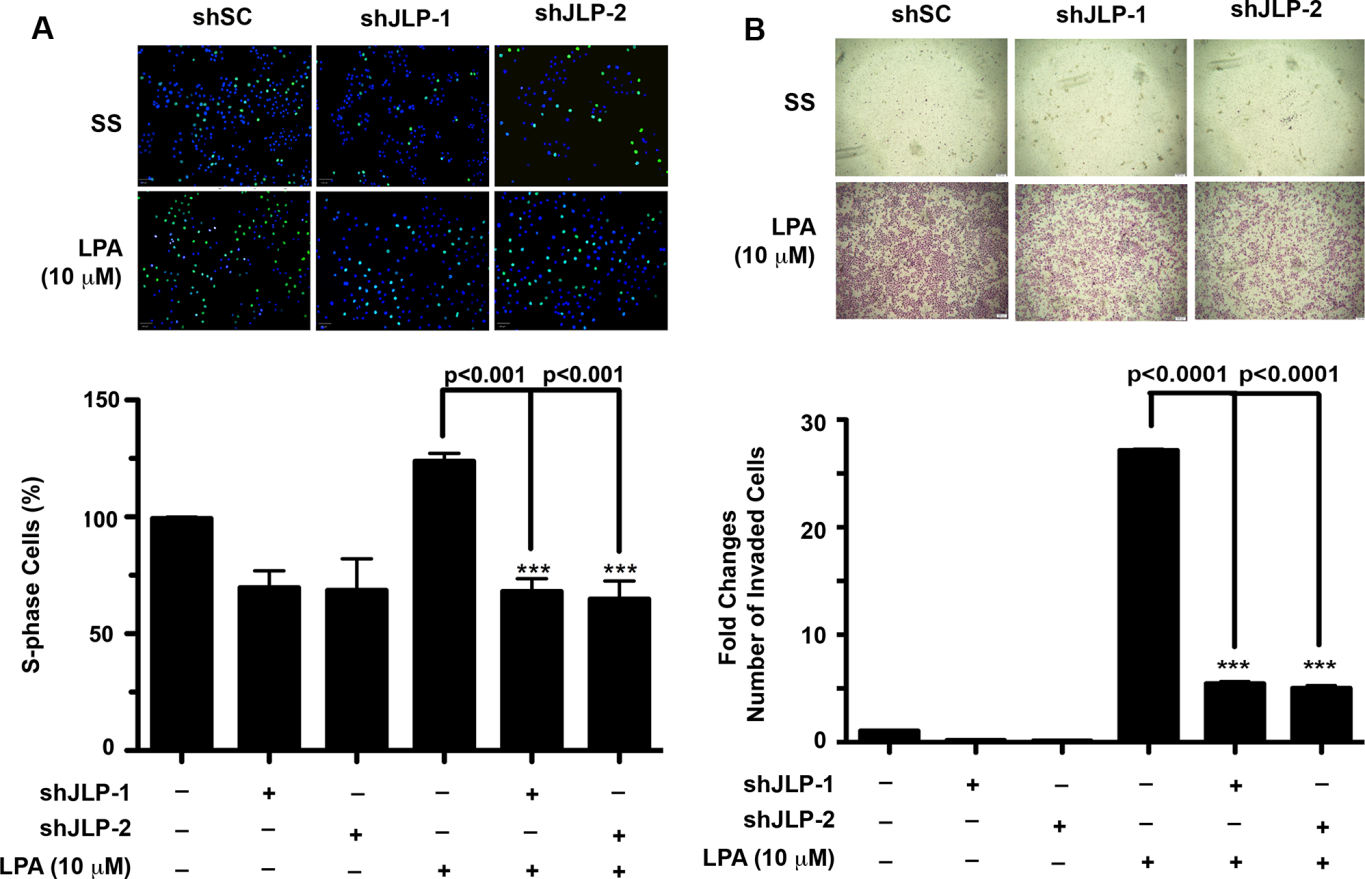
Knockdown of JLP attenuates LPA-stimulated proliferation and invasive migration (**A**) JLP-knockdown attenuates LPA-stimulated proliferation of SKOV3-ip cells. JLP-knocked down SKOV3-ip cells along with scrambled shRNA-expressing control cells (shSC) were plated in a 96-well plate (5 × 10^3^ cells/well). Serum starved cells were stimulated with 10 μM LPA along with appropriate control were incubated with EdU. EdU-incorporated proliferating cells versus the Hoechst33342-labeled total number of cells were imaged in the Operetta high-content imaging system (Upper Panel) and quantified (Lower Panel). Results are presented as percent change over the control values (Mean ± SEM; *n* = 3). (**B**) JLP-knockdown attenuates LPA-induced invasive migration of SKOV3-ip cells. Two independent clones of SKOV3-ip cells stably silenced for JLP and control cells pretreated with mitomycin-C were assayed for invasive migration using collagen-coated Transwell assay. Hemacolor-stained inserts were imaged at 10× magnification (Upper Panel). Cells migrated across the insert membrane were counted in five fields from three independent experiments (Mean ± SEM; *n* = 3). Results are presented as fold changes in the number of invaded cells over the control values (Lower Panel).

It has been shown by others and us that LPA acts as an aberrant growth factor that stimulates both proliferation and migration in ovarian cancer cells [22–24]. Therefore, we investigated whether JLP is required for LPA-stimulated oncogenic responses in ovarian cancer cells. First, we tested whether the silencing of JLP had any effect on cell proliferation. Cell proliferation was determined by monitoring the incorporation EdU into DNA as described above. SKOV3-ip cells, incubated with EdU were stimulated with LPA and the EdU-incorporated proliferating cells were imaged and quantified. As shown in Figure 4A, the silencing of JLP led to 40% reduction in S-phase cells. Studies from several laboratories including ours have established that LPA potently stimulates the invasive migration of ovarian cancer cells [22, 23]. It has also been shown that the activation of JNK is required for the migration of ovarian cancer cells [28–30]. Therefore, it can be reasoned that LPA stimulated invasive migration requires JLP presumably through the activation of JNK. To assess, SKOV3-ip cells in which the expression was silenced along with the controls cells expressing scrambled shRNA were stimulated with 10 μM LPA. Invasive migrations of these cells were monitored using collagen-coated transwell assay. Our results indicated that the silencing of JLP attenuated LPA-induced invasive migration by 42% (Figure 4B).

### Knockdown of JLP reduces ovarian xenograft tumor growth

**Figure 5:**
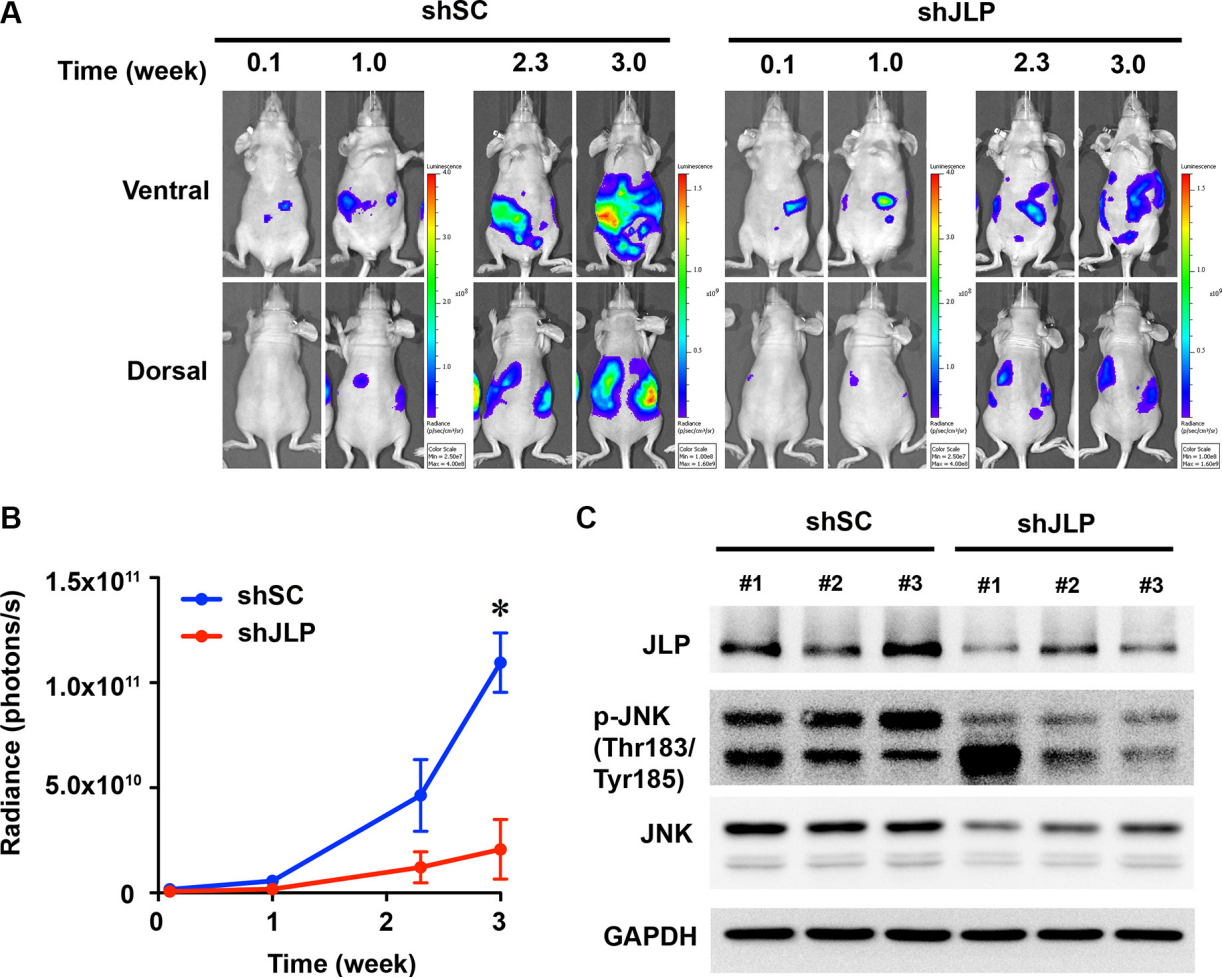
Knockdown of JLP represses ovarian xenograft tumor growth (**A**) JLP-knockdown represses ovarian xenograft tumor growth. JLP-silenced SKOV3-ip^Luc^shJLP or scrambled-shRNA SKOV3-ip^Luc^shSC control cells were intraperitoneally injected into nu/nu nude mice as described under Materials and Methods. The progression of injected cells was monitored by *in vivo* bioluminescence on an IVIS Spectrum imaging system. (**B**) Quantification of xenograft tumor growth. Tumor growth was monitored by increase in the radiance. The radiance of the bioluminescence was quantified as photons per seconds from four animals per group. The “*” mark denotes the euthanasia of the animals due to aggressive growth of xenograft tumors in control animals. (**C**) JLP-knockdown attenuates JNK-activation *in vivo*. Xenograft tumor tissues derived from JLP-silenced and control xenograft tumors were processed to monitor the expression as well as the activation profile of JNK using antibodies that recognize full-length JNK and antibodies that recognize phosphorylated JNK respectively. The blot was also probed with JLP-antibodies to monitor the efficacy of JLP-knockdown in JLP-silenced xenograft tumor tissues. The blot was stripped and re-probed for GAPDH-levels for monitoring equal protein loading.

Our results, presented thus far, have established a critical role for JLP in two critical oncogenic pathways, namely, cell proliferation and invasive migration. Both of these pathways contribute significantly to tumorigenesis and/or tumor progression in many cancers including ovarian cancers. Therefore, we investigated the role of JLP in ovarian cancer growth *in vivo* by using JLP-silenced xenograft tumor mouse model. To carry out such an analysis, we constructed luciferases-expressing bioluminescent JLP-silenced SKOV3-ip^Luc^-shJLP and non-effective scrambled shRNA encoding SKOV3-ip^Luc^shSC cell lines. SKOV3-ip^Luc^-shJLP or SKOV3-ip^Luc^shSC were inoculated intraperitoneally into female nu/nu mice and tumor growth was monitored every week by bioluminescence imaging in an IVIS spectrum imaging system. *In vivo* imaging of the xenograft tumor animals indicated that the silencing of JLP drastically reduced the tumor volume (Figure 5A). In addition, the imaging of the xenograft tumor also indicated that the silencing of JLP markedly reduced the aggressive intraperitoneal spread of ovarian cancer that could be observed in control animals (Figure 5A). Quantification of tumor volume based on the radiance of bioluminescence indicated that the tumor volume was significantly reduced in the JLP-silenced xenograft bearing mice (Figure 5B). It was also observed that the silencing of JLP prolonged the survival of these mice beyond 3 weeks compared to control animals bearing SKOV3-ip^Luc^shSC tumors (Figure 5B). Next, we examined whether the silencing of JLP had an inhibitory effect on the activation profile of JNK in the xenograft tumor tissues from these animals. Equally weighed and multiple tissue excisions of the xenograft control and tumor tissues were homogenized, and the tissue lysates were subjected to immunoblot analysis to monitor the expression levels of JNK. The activation profile of JNK was monitored using antibodies against phosphorylated JNK. Our results indicated that the JLP-silenced xenograft tumors showed reduced levels of JLP as anticipated (Figure 5C). A similar reduction in the levels of phosphorylated JNK was observed in JLP-silenced xenograft tumor tissues compared to control xenograft tumor tissues. JLP-silenced xenograft tumor tissues also showed an overall reduction in the expression levels of JNK. Although the mechanism underlying the reduced expression of JNK is not fully understood at present, the correlation between the silencing of JLP and the reduced levels of JNK/phospho-JNK levels clearly points to JNK as a potential mechanism underlying the less aggressive growth of tumor xenografts in the mice.

**Figure 6:**
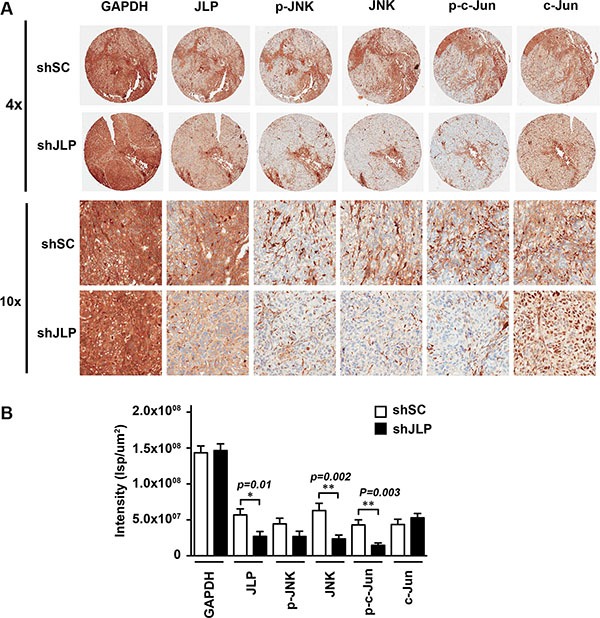
Knockdown of JLP attenuates the activation of JNK and c-Jun in ovarian cancer xenografts (**A**) Immunohistochemical analysis of xenograft tumor tissue. A TMA, constructed using the tissues derived from JLP-silenced xenograft tumors along with the xenografts expressing scrambled shRNA, was processed for immunohistochemical analysis using antibodies to JLP, activated JNK (p-JNK), JNK, activated c-Jun (p-c-Jun), and c-Jun. Representative cores of the TMA for each of the antibodies at 4×- (Upper Panel) and 10×-magnifications (Lower Panel) are presented. (**B**) Average staining intensities for each of the protein derived from a random nine fields (*n* = 3 animals; Mean ± SEM) were quantified by Spectrum software (Aperio, Vista, CA) and the results were expressed as the Intensity of strong positive (Isp) per square micrometer.

To further characterize the functional alterations in the JLP-JNK signaling node in these xenograft tumors, we monitored the activation profile of c-Jun in the JLP-silenced xenograft tumors. To analyze, a TMA was constructed with an equal distribution of control and JLP-silenced xenograft tumor tissues. The TMA was subjected to IHC analyses using appropriate antibodies to monitor the expression of JLP, JNK, phospho-JNK, c-Jun, and phospho-c-Jun. Results from such IHC analysis confirmed that the JLP-silenced ovarian tumor xenografts exhibited reduced expression of JLP with the concomitant reduction in the expression levels of JNK as well as its phosphorylated activation profile (Figure 6A). More strikingly, these tumors showed a drastic reduction in the phosphorylation status of c-Jun with little or no change in its expression levels (Figure 6A). Quantification of the results indicated that the phosphorylation of c-JUN was reduced in these tumors by 65% (Figure 6B). These findings, together with our results from the analyses of xenograft tumor growth and intraperitoneal tumor spread (Figure 6), establish that the silencing of JLP with the resultant decrease in the activation of JNK signaling node inhibits tumor growth and intraperitoneal spread of ovarian xenograft tumors.

## DISCUSSION

Scaffold proteins for MAPKs form a functionally conserved family of proteins that present across species from yeast to humans [1]. Scaffold proteins have been shown to provide a physical framework for the organization of the three-tier kinase modules such as those of ERKs, JNKs, and p38MAPKs. While the role of the individual MAPKs in cancer etiology and progression has been realized to a great extent [1, 3], the potential role of the interacting scaffold proteins in promoting tumorigenesis is largely unknown. In this context, our findings that the coexpression of JLP, a JNK-interacting scaffold protein, enhanced the tumorigenic potential of the *gep* oncogenes provided an early indication that JLP could be involved in promoting tumorigenesis and tumor progression [7, 8]. Our studies presented here present the first evidence that the expression of JLP is increased in ovarian cancer cell lines as well as patient-derived ovarian cancer cells and tissue (Figure 1). Using specific antibodies that only recognize the N-terminal epitope of JLP, which is absent in SPAG9 and JIP4, our studies unequivocally demonstrate the overexpression of JLP in multiple ovarian cancer cells and cancer tissue. By ectopically overexpressing JLP in SKOV3-ip cells, we could demonstrate that the increased expression of JLP promoted the invasive migration of these cells (Figure 2). Our results presented in Figure 3 along with our previous findings [7, 8] demonstrate further that JLP and JNK physically interact with each other and this interaction is stimulated by LPA, a pathophysiological ligand that promote ovarian cancer cell proliferation and metastasis

Interrogating further, we demonstrate here that LPA stimulates the colocalization of JLP and JNK and subsequent translocation of JLP-JNK complex to nuclear periphery from where the phosphorylated JNK could enter into the nucleus (Figure 3). Silencing the expression of JLP in these cells abrogates LPA-stimulated activation of JNK by 68% (Figure 3B). More interestingly, overexpression of JLP, in addition to activating JNK, promotes the invasive migration of ovarian cancer cells (Figure 2C). Pathophysiological significance to this data is exemplified by the findings that the silencing of JLP also mitigated LPA-stimulated invasive migration of SKOV3-ip ovarian cancer cells (Figure 4B). These findings gain greater pathological significance considering the elevated levels of LPA present in the serum and the ascites of ovarian cancer [31, 32]. It can be reasoned that the higher concentrations of LPA in ovarian cancer, in conjunction with the increased expression of JLP in ovarian cancers can stimulate intraperitoneal metastatic invasive migration of ovarian cancer cells and promotes aggressive tumor growth. In fact, our analysis of the role of JLP using SKOV3-ip-based xenograft model in which the silencing of JLP abrogates xenograft tumor growth and intraperitoneal spread of the disease (Figure 5A) validates this conclusion. Consistent with our conclusion that this is due to the abrogated activation of JNK in these xenograft tumors, the tumor-derived tissue samples showed a reduction in the expression as well as activation of JNK (Figures 5 and 6) along with a decrease in the levels of activated phosphorylated c-Jun (Figure 6B). The observation that the JLP-silenced xenograft tumors show a reduction in the expression levels of JNK was rather unanticipated. Interestingly, this correlation between the expression levels of JLP and JNK is being observed only in the xenograft tumor model and not in cell based expression analysis. Although the underlying mechanism is not clear at present, it is possible that JLP somehow stabilizes the expression levels of JNK and the knockdown of JLP alters this equilibrium. The observation that the brain and pancreatic β-cell specific JNK1-scaffold protein IB1/JIP1 enhances the stability of JNK1 levels attests to this possibility [33]. While further studies are needed to firmly establish this point, our results do point out that the knockdown of JLP negatively affects both the expression levels and the activation profile of JNK *in vivo* presumably contributing to the reduction in the phosphorylated activation profile of downstream c-Jun. Finally, using SKOV3-ip cell based xenograft model of ovarian cancer, we demonstrate here for the first time, that the aberrant expression of JLP can greatly contribute to the aggressive growth and intraperitoneal spread of ovarian cancer.

Identification of critical nodes involved in tumor progression is of critical importance in developing newer therapies for cancer. In this regard, defining the role of JNK-specific scaffold protein has become important in light of the observation that JNK-module is involved in both growth-promoting as well as apoptotic-inducing pathways [1, 34]. Recent findings have identified the pro-tumorigenic role JNK-signaling module in many cancers [21, 35–37] in addition to establishing the efficacy of JNK inhibitors in suppressing cancer cell growth *in vitro* and *in vivo* [20, 38–40]. These results together with our findings that the silencing of JLP leads to a reduction in xenograft tumor growth and the intraperitoneal spread of the disease identifies a hitherto unknown function for JLP in providing a pro-tumorigenic physical conduit for JNK-signaling pathway. Studies in yeast have shown that the scaffold proteins play a major role in context specific signaling [41, 42]. For instance, the scaffold protein Ste5p provides scaffold for the transmission of signals from Ste2 pheromone-receptor to Ste20p-Ste11p-Ste7p kinase module in eliciting mating response in yeast [1, 41–44]. However, the starvation signals bypasses Ste5 to activate the same kinase module to elicit filamentation response [44, 45]. Similarly, an osmotic shock signal in yeast recruits the Pbs2, a MAP2K, which also serves as a scaffold in assembling kinases module Ste20 and Ste7 to elicit osmo-adaptive response [45]. Thus, using the same complement of kinases, distinct scaffold proteins channelize the signals from the kinases to elicit specific cellular responses in yeast. Based on these observations it can be envisaged that JLP directs JNK-signaling to oncogenic cellular response. This is also supported by the previous finding that JIP4, the 3.4 kbp splice variant of *SPAG9*, has been characterized to be involved only in assembling p38MAPK-module but not that of JNK [10]. Although our present findings point to a role for JNK in JLP-mediated oncogenic signaling, it is possible that the other JLP-interacting proteins such as PLK1 can also have a significant role in JLP-promoted oncogenic signaling [46]. Further studies should define the interrelationship among these JLP-binding partners.

**Figure 7:**
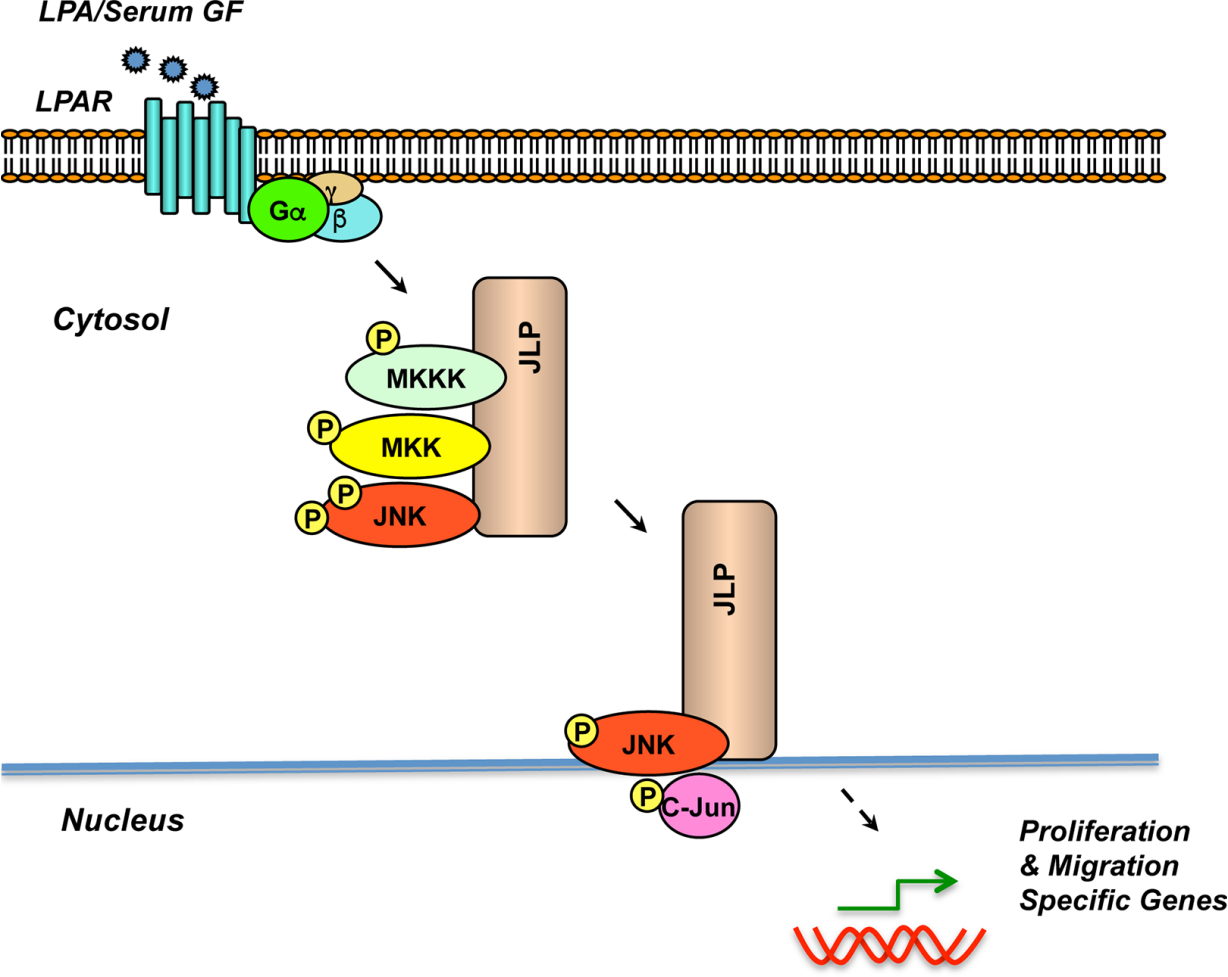
Schematic Representation of the role of JLP in ovarian cancer progression Results presented here along with the previous findings [6–9] suggest a model in which LPA stimulates the assembly of JLP scaffold involving JNK-signaling module. Upon stimulation with LPA, LPA-receptor (LPAR) stimulates the cognate G protein and its interaction with JLP. JLP, in turn, tethers the JNK-signaling module consisting of MAP kinase kinase kinase (MKKK), MAP kinase kinase (MKK4/7) and Jun kinase (JNK). Such an assembly promotes accelerated signal propagation from JNK to c-Jun as well as other JNK-target transcription factors, presumably through the observed JLP-mediated translocation of JNK to nuclear periphery. Resultant transcriptional activation of proliferation and migration specific genes contributes to the aggressive growth and intraperitoneal spread of ovarian cancer.

Together with the previous observations that the *gep* proto-oncogenes that are stimulated by LPA play a determinant role in ovarian cancer progression and they physically associate with JLP [7, 8], our present results point to a signaling paradigm in which JLP plays a scaffolding role in transmitting the signals from LPA to an oncogenic response (Figure 7). Our findings gain further significance from cBioPortal (http://www.cbioportal.org) analysis of TCGA datasets of ovarian carcinoma cancer patients [17–19]. Although the amplification of SPAG9/JLP was observed only in 3 % of the cases analyzed, the increased expression correlates with the decreased disease-free survival of ovarian cancer patients (Supplementary Material S2). Thus, it is reasonable to conclude that the hyper-stimulation of JLP by LPA in ovarian cancer cells could lead to a similar outcome in the patients. It is well documented that LPA is present in an alarmingly high concentrations in the serum as well as the ascites of ovarian cancer patients [32]. This is primarily due to the increased synthesis of LPA by the ovarian cancer cells. Thus, LPA, which is present in the ascites, could elicit a persistent hyper-activation of JLP-scaffold and its associated kinase module. This, in turn could contribute significantly to the aggressive progression of ovarian cancer - even in the absence of SPAG9/JLP gene amplification. Thus, in addition to unraveling the critical role of JLP in ovarian cancer pathobiology, our studies identify the JLP-signaling node as a valuable therapeutic target.

## MATERIALS AND METHODS

### Cell lines

SUN119, ES-2, OV90, CaOV3, TOV21G, and the patient-derived cell line A39 were from Seoul National University, Seoul, Korea. Immortalized normal ovarian epithelial cells (OSE), fallopian-tube-derived epithelial cells (FTE188), and SKOV3-ip cell lines have been previously described and used [47–49]. OVCAR4 cell line was obtained from Dr. Thomas Hamilton (Fox Chase Cancer Center, PA). Kuramochi, TYKNU, and OVSAHO cell lines were from the JCRB Cell Bank, Tokyo, Japan while COV362, OAW28 and COV318 cells were purchased from Sigma-Aldrich (St. Louis, MO). Cell-culture conditions and the construction of bioluminescent JLP-silenced SKOV3-ip^Luc^-shJLP and non-effective scrambled shRNA-expressing SKOV3-ip^Luc^shSC cell lines are detailed in Supplementary Material S1. Transient and stable transfections were carried out using a Nucleofector II from Lonza (Allendale, NJ) as previously described [50, 51].

### Immunohistochemical staining

Immunohistochemistry was performed according to manufacturer's protocol using Leica Bond™ Polymer Refine Detection system (DS 9800). In brief, formalin-fixed paraffin-embedded tissues were sectioned (4 μm) and mounted on positively charged slides. The slides were dried overnight at room temperature and incubated at 60°C for 45 minutes followed by deparaffinization and rehydration in an automated Multistainer (Leica ST5020). Subsequently, these slides were transferred to the Leica Bond-III™, treated for target retrieval at 100°C for 20 minutes with a retrieval buffer, pH 6.0. Endogenous peroxidase was blocked using peroxidase-blocking reagent, followed by the primary antibody, SPAG9N (Aviva OAAB04262; 1:50 dilution) incubation. For the secondary antibody, post-primary IgG-linker and/or Poly-AP IgG reagents were used. The substrate chromogen, 3, 3′-diaminobenzidine tetrahydrochloride (DAB) detects the complex as brown precipitate, while hematoxylin counterstain the cell nuclei (blue). The slides were then dehydrated (Leica ST5020), and mounted (Leica MM24). Antibody specific positive and negative (omission of primary antibody) controls were parallel stained.

### IHC of ovarian cancer tissue

IHC was carried out with appropriate controls on the normal and ovarian cancer tissues and they were evaluated using the H-score. The staining intensity was graded using a 0–3+ scale with 0 being no appreciable staining to 3+ being strong staining. The percentage of cells stained along with the grade of staining was assessed at two different magnifications. The following are the observed data using the ovarian cancer tissue sample:

Control × 20: 0, 0%

Control × 60: 0, 0%

Ovarian Cancer Sample × 20: 3+, 40%; 2+, 30%; 1+, 30%

Ovarian Cancer Sample x 60: 3+, 60%; 2+, 20%; 1+, 20%

Final score was calculated using the following H-score formula:

[1 × (% cells 1+) + 2 × (% cells 2+) + 3 × (% cells 3+)].

### Generation of xenografts and bioluminescence imaging

SKOV3-ip^Luc^shSC or SKOV3-ip^Luc^shJLP cells (5 × 10^6^ in 0.2 mL PBS) were intraperitoneally injected into the lower right quadrant of 1.5-month old female nu/nu nude mice (Charles River, Wilmington, MA). The progression of injected cells was monitored by *in vivo* bioluminescence on an IVIS Spectrum imaging system (PerkinElmer, Waltham, MA). Intraperitoneal injection of D-Luciferin (PerkinElmer) was administered to the isofluorane-anesthetized mice at 150 mg/kg of body weight. After 10 minutes, the mice were re-anesthetized and the bioluminescent images were taken at both ventral and dorsal positions. The radiance of the bioluminescence was quantified as photons per second. After euthanasia, the xenograft tumors were excised, fixed in 10% formalin, and used to create the tumor tissue microarray (TMA). TMA was constructed at the Cancer Tissue Pathology Core of the Stephenson Cancer Center. IHC staining was carried out as described above on a BOND-III automated system (Leica, Buffalo Grove, IL). Intensity of strong positive (Isp) per square micrometer of the IHC image was quantified using Spectrum software (Aperio, Vista, CA).

### Cell proliferation assay

Proliferation was monitored by determining the percentage of proliferating S-phase positive cells using the deoxynucleotide (5-ethynyl-2′-deoxyuridine or EdU) incorporation assay (Click-iT Plus EdU Alexa Fluor 488 imaging kit: C10637, Life Technologies, Grand Island, NY). The labeled fluorophores were imaged and quantified in Operetta high-content imaging system (PerkinElmer).

### Colony formation/clonogenic assay

This assay was carried out following the previously published procedures [27]. Immortalized normal FTE188 were transfected with pcDNA3-vector or vector encoding JLP using Nucleofector II system. The transfectants were plated at a density of 1000 cells per 35 mm plate in MEM and grown for 12 days. Colonies were fixed and visualized by staining with 0.2% crystal violet. The stained colonies were quantified using automated GelCount Colony Counter (Oxford Optronix, Abington, UK).

### Invasive cell migration assay

Migration assays were performed using a 24-well Boyden chamber with an 8 μm pore size transparent polyethylene terephthalate (PET) membrane (#353097, Corning) coated with collagen type I (BD Biosciences, San Jose, CA). SKOV3-ip cells were serum starved overnight and added to the upper chamber at 5 × 10^4^ cells per chamber, and indicated chemoattractant was added to the lower chamber in serum-free RPMI 1640 media. After incubation for 20 h at 37°C, the cells on the upper surface of the membrane were gently removed, and the cells on the lower surface were fixed and stained with Hemacolor (EMD Millipore, Billerica, MA). Images of the entire lower surface of the membranes were taken, and the number of migrated cells was counted (four wells per condition).

### Immunofluorescence imaging

SKOV3-ip cells were plated on 96-well plate and stimulated with 10 μM LPA, fixed with 3% paraformaldehyde in PBS for 10 minutes, permeabilized with 0.1% Triton-X-buffer for 10 minutes and blocked for 30 minutes with ice cold 0.1% BSA in PBS. Sequential immunostaining was carried out with primary mouse monoclonal JNK (1:200) and rabbit JLP (1:100) antibody for 1h, washed, incubated with secondary Alexa 488-conjugated goat anti-mouse IgG (1:500) and Alexa 568-conjugated goat anti-rabbit IgG (1:500) for 1h and washed with 1x TBST. Nuclei were counter stained with DAPI (diamidino-2-phenylindole, 1 μg/mL) for 5 minutes and washed finally with PBS and optical imaging, colocalization assessment were carried out in Operetta high-content imaging system.

### Immunoprecipitation and immunoblot analysis

Co-immunoprecipitation analyses were carried out according to our previously published methods [47, 51, 52]. Immunoblot analysis for JNK, pJNK, JLP, GAPDH, and with the indicated antibodies were carried out following previously published procedures [47, 51, 52] and developed with a Kodak Image Station 4000 MM.

### Statistical analysis

All statistical analysis was performed using GraphPad Prism (La Jolla, CA) by two-tailed student's *t-test* with Welch's correction.

## SUPPLEMENTARY MATERIALS FIGURE


