# Supplementary material for: Aberrant expression of JNK-associated leucine-zipper protein, JLP, promotes accelerated growth of ovarian cancer
Source: Oncotarget. 2016 Sep 16;7(45):72845–59. doi: 10.18632/oncotarget.12069 (PMC5341948; doi:10.18632/oncotarget.12069)
Supplement: Supplementary file 1 [file oncotarget-07-72845-s001.pdf]

Aberrant expression of JNK-associated leucine-zipper protein, JLP, promotes accelerated growth of ovarian cancer

Supplementary Materials

Cell culture conditions

SNU119, OVCAR4, SKOV3-ip, OVSAHO, and Kuramochi cell lines were cultured in RPMI 1640 (Mediatech, Manassas, VA); COV362, COV318, and CaOV3 cells were cultured in DMEM; TYKNU cells were cultured in EMEM (Corning); FTE188, TOV21G, OSE, and OV90 cells were maintained in Media 199/ MCDB 205 (1:1; Sigma-Aldrich Co.); and ES-2 cells were cultured in McCoy's 5A Medium. All media were supplemented with 10% fetal bovine serum (Atlanta Biologicals, Flowery Branch, GA), 100 U/ml penicillin, and 100 μg/ml streptomycin (Mediatech) in a humidified atmosphere of 5% CO_2 and 95% air at 37°C. Construction of bioluminescent JLP-knockdown cells.

Cell proliferation assay

The assay was carried out following previously published procedure. Briefly, 5 × 103 cells of each cell line were plated in 96 well plates, serum starved, stimulated with 10 μM LPA and incubated with 10 μM EdU at 22 hours. After 2 hours of incubation, the EdU incorporated cells were fixed, permeabilized, and the ethynyl-moeity of EdU was labeled with azide-coupled Alexa Fluor 488 according to the manufacturer's protocol. Proliferating cells versus the total number of Hoechst 33342-labeled cells were quantified in Operetta High Content Imaging System using the Harmony image analysis software (PerkinElmer, Waltham, MA). Results are presented as percent change over the respective control values.

The construction of bioluminescent SKOV3-ip^{Luc} cells

JLP-silenced SKOV3-ip^{Luc}-shJLP and non-effective scrambled shRNA encoding SKOV3-ip^{Luc}shSC cell lines, was carried out by engineering an expression-cassette encoding cell culture verified unique 29-mer shRNA targeting JLP (5′-CATCGAATCTACTCCTGAGCTGGATATGG-3′) or non-effective 29-mer scrambled control shRNA (5′-GCACTACCAGAGCTAACTCAGATAGTACT-3′) into an pGL4.51 expression vector encoding neomycin resistance and codon-optimized luciferase reporter (Promega, Madison, WI). U6-promoter driven control or JLP-targeting shRNA cassettes, were excised from pRS constructs (# TR30012 and TI305760 respectively from Origene, Rockville, MD) using NaeI and NsiI and ligated into BamHI (5′ overhang filled in by Klenow) and NsiI sites of the pGL4.51 vector to generate control pGL-shSC or JLP-targeting pGL-shJLP vectors. SKOV3-ip cells were transfected with PstI-linearized pGL-shSC or pGL-shJLP by Amaxa Nucleofector (Lonza, Basel, Switzerland) and selected in the presence of 1 mg/mL G418 sulfate (EMD Millipore, Billerica, MA). The derivative monoclonal stable cell lines SKOV3-ip^{Luc}shSC and SKOV3-ip^{Luc}shJLP stable were isolated and propagated. Knockdown of JLP in the transfectants was verified by immunoblot analysis.

Antibodies used in the study

JLP-antibodies (5519) as well as antibodies to phospho-JNK (9251) and b-actin (8437) for immunoblot analyses were obtained from Cell Signaling Technology (Danvers, MA). JNK-antibodies (06-748) for immunoprecipitation and GAPDH-antibodies for immunoblot analysis (CB1001) were purchased from EMD Millipore (Billerica, MA) whereas JNK-antibodies (SC-7345) for immunoblot analysis and immunolocalization were obtained from Santa Cruz Biotechnology (Santa Cruz, CA). Alexa 568 anti-mouse and Alexa 488 anti-rabbit were purchased from Invitrogen (Eugene, OR). DAPI was purchased from Life Technologies and used at a working concentration of 0.25 μg/ml. Peroxidase-conjugated anti-rabbit IgG was purchased from Promega (Madison, WI), and peroxidase-conjugated anti-mouse was purchased from GE Healthcare (Little Chalfont, UK).

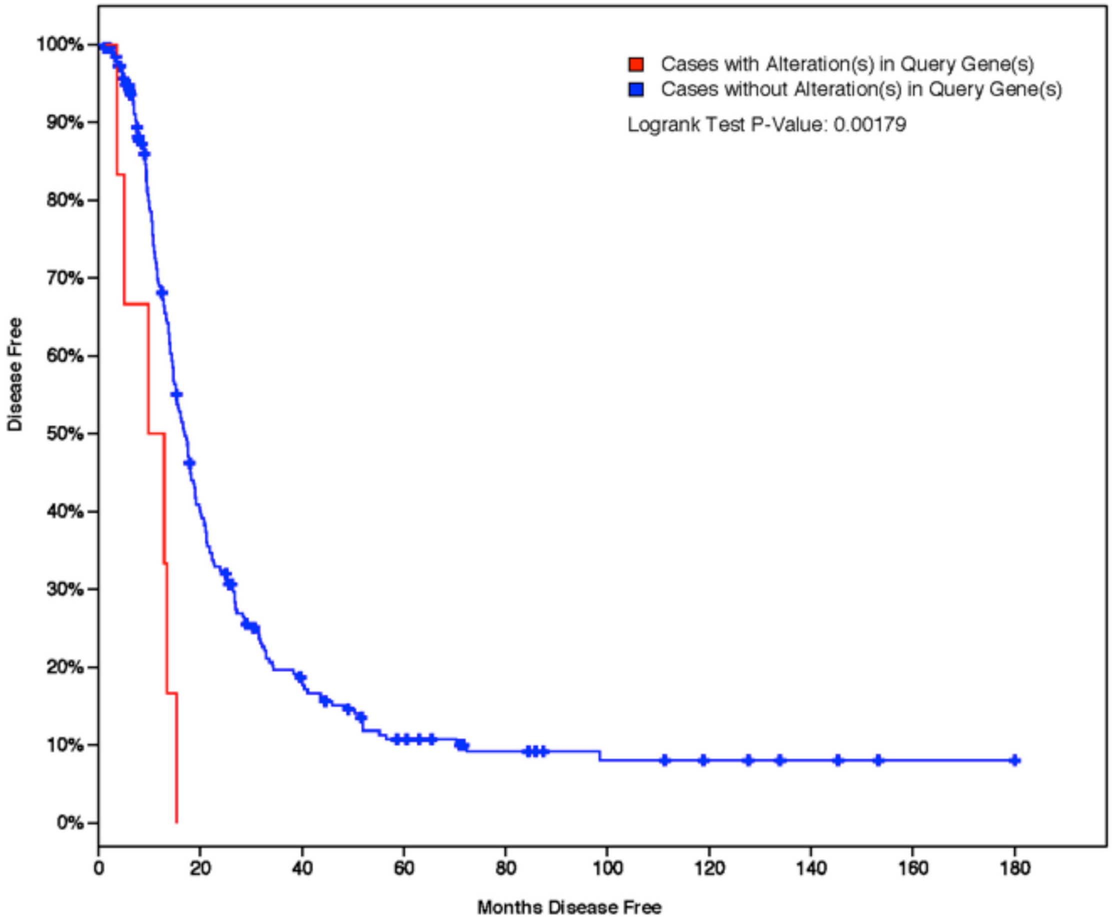

Supplementary Figure S1: Amplification of *SPAG9/JLP* gene reduces DFS of ovarian cancer patients. Kaplan–Meier plot of disease free survival associated with JLP overexpression in ovarian cancer patients. This plot is obtained from cBioPortal (http://www.cbioportal.org) analysis of the data from the TCGA database (18, 19). The amplification of *SPAG9/JLP* gene is observed only in 3% of the analyzed ovarian cancer patient samples in TCGA dataset (20). However, the median months of disease free survival (DFS) is reduced by 42% in these patients. Red line denotes gene alterations in *SPAG9/JLP* whereas blue line indicates the absence of any such change.
